# Supplementary material for: Sex differences in growth and mortality in pregnancy-associated hypertension
Source: PLoS One. 2024 Jan 11;19(1):e0296853. doi: 10.1371/journal.pone.0296853 (PMC10783718; doi:10.1371/journal.pone.0296853)
Supplement: S2 Table — (DOCX) [file pone.0296853.s002.docx]

S2 Table. Effects on birthweight among infants who were still alive at 29 days after birth among White (N = 5122926) and Black (N = 1089012) individuals.

| Effect |  | White | | Black | |
| --- | --- | --- | --- | --- | --- |
|  | Numerator DF | F value | p value | F value | p value |
| Gestational age (categorical) | 18 | 79230 | < 0.0001 | 23441 | < 0.0001 |
| Sex | 1 | 716.05 | < 0.0001 | 370.03 | < 0.0001 |
| Group (PAH / control) | 1 | 2007.07 | < 0.0001 | 986.39 | < 0.0001 |
| Tobacco use (Y / N) | 1 | 110007 | < 0.0001 | 4861.66 | < 0.0001 |
| Year | 11 | 49.24 | < 0.0001 | 25.52 | < 0.0001 |
| Gestational age * Sex | 18 | 18.58 | < 0.0001 | 6.72 | < 0.0001 |
| Gestational age * Group | 18 | 605.74 | < 0.0001 | 206.29 | < 0.0001 |
| Sex * Group | 1 | 0.57 | 0.45 | 0.49 | 0.48 |
| Gestational age * Sex * Group | 18 | 5.06 | < 0.0001 | 1.06 | 0.39 |
