# Supplementary material for: Sex differences in growth and mortality in pregnancy-associated hypertension
Source: PLoS One. 2024 Jan 11;19(1):e0296853. doi: 10.1371/journal.pone.0296853 (PMC10783718; doi:10.1371/journal.pone.0296853)
Supplement: S3 Table — (DOCX) [file pone.0296853.s003.docx]

S3 Table. Effects on birthweight, including fetal deaths and infants who died within 28 days of birth among White (N = 5143779) and Black (N = 1097178) individuals.

| Effect |  | White | | Black | |
| --- | --- | --- | --- | --- | --- |
|  | Numerator DF | F value | p value | F value | p value |
| Gestational age (categorical) | 18 | 26027 | < 0.0001 | 10127 | < 0.0001 |
| Sex | 1 | 746.88 | < 0.0001 | 164.46 | < 0.0001 |
| Survived (Y / N) | 1 | 3693.83 | < 0.0001 | 1240.23 | < 0.0001 |
| Group (PAH / control) | 1 | 6139.01 | < 0.0001 | 2800.15 | < 0.0001 |
| Tobacco use (Y / N) | 1 | 109177 | < 0.0001 | 4767.78 | < 0.0001 |
| Year | 11 | 51.98 | < 0.0001 | 28.32 | < 0.0001 |
| Gestational age * Sex | 18 | 5.38 | < 0.0001 | 3.08 | < 0.0001 |
| Gestational age * Survived | 18 | 40.46 | < 0.0001 | 21.59 | < 0.0001 |
| Sex * Survived | 1 | 12.79 | 0.0003 | 19.67 | < 0.0001 |
| Gestational age * Sex * Survived | 18 | 0.52 | 0.95 | 1.22 | 0.23 |
