# Supplementary material for: Sex differences in growth and mortality in pregnancy-associated hypertension
Source: PLoS One. 2024 Jan 11;19(1):e0296853. doi: 10.1371/journal.pone.0296853 (PMC10783718; doi:10.1371/journal.pone.0296853)
Supplement: S4 Table — (DOCX) [file pone.0296853.s004.docx]

S4 Table. The odds of death for males relative to females in PAH pregnancies and controls in White pregnancies. An OR > 1 indicates that males are at greater risk of death than females within a group. A sliding window was used to analyse the odds of death at that week or in the following two weeks.

| Gestational age | PAH Male  Surviving | PAH Male Died | PAH Female  Surviving | PAH Female Died | Control Male  Surviving | Control Male Died | Control Female Surviving | Control Female  Died | P-values from logistic regression | | | Odds ratio for death in males vs. females among PAH pregnancies | | | Odds ratio for death in males vs. females among Control pregnancies | | | |
| --- | --- | --- | --- | --- | --- | --- | --- | --- | --- | --- | --- | --- | --- | --- | --- | --- | --- | --- |
|  | N | N | N | N | N | N | N | N | Sex | Group (PAH vs Control) | Sex*Group | Estimate | Lower CL | Upper CL | Estimate | | Lower CL | Upper CL |
| 24 | 188506 | 182 | 167979 | 199 | 2942122 | 2049 | 2797907 | 1645 | 0.7426 | <.0001 | 0.0005 | 0.815 | 0.666 | 0.996 | | 1.185 | 1.110 | 1.264 |
| 25 | 188445 | 191 | 167916 | 201 | 2941537 | 1832 | 2797505 | 1388 | 0.5751 | <.0001 | 0.0002 | 0.846 | 0.694 | 1.031 | | 1.255 | 1.171 | 1.346 |
| 26 | 188352 | 211 | 167852 | 190 | 2940925 | 1823 | 2797062 | 1346 | 0.0224 | <.0001 | 0.0131 | 0.990 | 0.813 | 1.204 | | 1.288 | 1.200 | 1.382 |
| 27 | 188275 | 231 | 167797 | 182 | 2940520 | 1602 | 2796716 | 1191 | 0.0005 | <.0001 | 0.2470 | 1.131 | 0.931 | 1.374 | | 1.279 | 1.187 | 1.379 |
| 28 | 188199 | 246 | 167747 | 169 | 2940074 | 1463 | 2796373 | 1132 | <.0001 | <.0001 | 0.6147 | 1.298 | 1.067 | 1.578 | | 1.229 | 1.137 | 1.328 |
| 29 | 188096 | 217 | 167642 | 151 | 2939150 | 1280 | 2795624 | 982 | <.0001 | <.0001 | 0.7748 | 1.281 | 1.041 | 1.577 | | 1.240 | 1.141 | 1.347 |
| 30 | 187900 | 224 | 167436 | 143 | 2937806 | 1369 | 2794551 | 1006 | <.0001 | <.0001 | 0.5109 | 1.396 | 1.132 | 1.722 | | 1.294 | 1.193 | 1.404 |
| 31 | 187617 | 205 | 167116 | 136 | 2936149 | 1391 | 2793310 | 995 | <.0001 | <.0001 | 0.9359 | 1.343 | 1.081 | 1.668 | | 1.330 | 1.226 | 1.443 |
| 32 | 187224 | 203 | 166643 | 135 | 2934197 | 1436 | 2791689 | 1091 | <.0001 | <.0001 | 0.5735 | 1.338 | 1.077 | 1.664 | | 1.252 | 1.157 | 1.355 |
| 33 | 186585 | 185 | 166014 | 127 | 2931883 | 1479 | 2789902 | 1131 | <.0001 | <.0001 | 0.7382 | 1.296 | 1.034 | 1.625 | | 1.244 | 1.152 | 1.345 |
| 34 | 185748 | 203 | 165227 | 134 | 2929029 | 1657 | 2787755 | 1299 | <.0001 | <.0001 | 0.3697 | 1.349 | 1.084 | 1.678 | | 1.214 | 1.129 | 1.306 |
| 35 | 184723 | 196 | 164238 | 137 | 2925384 | 1875 | 2785034 | 1464 | 0.0002 | <.0001 | 0.7161 | 1.272 | 1.023 | 1.583 | | 1.219 | 1.139 | 1.306 |
| 36 | 183424 | 200 | 162997 | 128 | 2920389 | 2147 | 2781411 | 1748 | <.0001 | <.0001 | 0.1455 | 1.389 | 1.112 | 1.734 | | 1.170 | 1.098 | 1.246 |
| 37 | 181600 | 189 | 161368 | 121 | 2913108 | 2369 | 2775925 | 2011 | 0.0002 | 0.0190 | 0.0763 | 1.389 | 1.106 | 1.746 | | 1.123 | 1.058 | 1.191 |
| 38 | 178979 | 202 | 159026 | 133 | 2901263 | 2765 | 2766626 | 2460 | 0.0013 | 0.3477 | 0.0452 | 1.350 | 1.084 | 1.680 | | 1.072 | 1.015 | 1.132 |
| 39 | 174937 | 162 | 155404 | 132 | 2878701 | 2624 | 2748260 | 2403 | 0.2887 | 0.9131 | 0.7105 | 1.090 | 0.866 | 1.372 | | 1.042 | 0.986 | 1.102 |
| 40 | 168090 | 119 | 149608 | 106 | 2832447 | 2163 | 2710877 | 1985 | 0.7638 | 0.4283 | 0.7550 | 0.999 | 0.769 | 1.298 | | 1.043 | 0.981 | 1.108 |
| 41 | 154911 | 61 | 138593 | 50 | 2736809 | 1241 | 2631449 | 1120 | 0.4400 | 0.1167 | 0.9018 | 1.091 | 0.751 | 1.586 | | 1.065 | 0.983 | 1.155 |
| 42 | 131096 | 35 | 118304 | 22 | 2537108 | 651 | 2461194 | 560 | 0.0835 | 0.5613 | 0.3862 | 1.435 | 0.842 | 2.446 | | 1.128 | 1.007 | 1.263 |
