# Supplementary material for: Sex differences in growth and mortality in pregnancy-associated hypertension
Source: PLoS One. 2024 Jan 11;19(1):e0296853. doi: 10.1371/journal.pone.0296853 (PMC10783718; doi:10.1371/journal.pone.0296853)
Supplement: S5 Table — (DOCX) [file pone.0296853.s005.docx]

S5 Table. The odds of death for males relative to females in PAH pregnancies and controls in Black pregnancies. An OR > 1 indicates that males are at greater risk of death than females within a group. A sliding window was used to analyse the odds of death at that week or in the following two weeks.

| Gestational age | PAH Male  Surviving | PAH Male Died | PAH Female  Surviving | PAH Female Died | Control Male  Surviving | Control Male Died | Control Female Surviving | Control Female  Died | P-values from logistic regression | | | Odds ratio for death in males vs. females among PAH pregnancies | | | Odds ratio for death in males vs. females among Control pregnancies | | |
| --- | --- | --- | --- | --- | --- | --- | --- | --- | --- | --- | --- | --- | --- | --- | --- | --- | --- |
|  | N | N | N | N | N | N | N | N | Sex | Group (PAH vs Control) | Sex*Group | Estimate | Lower CL | Upper CL | Estimate | Lower CL | Upper CL |
| 24 | 34410 | 87 | 34351 | 101 | 602855 | 1213 | 580218 | 985 | 0.9010 | <.0001 | 0.0355 | 0.860 | 0.645 | 1.146 | 1.185 | 1.090 | 1.289 |
| 25 | 34374 | 94 | 34312 | 105 | 602561 | 1016 | 579960 | 839 | 0.7870 | <.0001 | 0.0756 | 0.893 | 0.676 | 1.180 | 1.166 | 1.064 | 1.277 |
| 26 | 34341 | 94 | 34274 | 107 | 602281 | 914 | 579738 | 760 | 0.9209 | <.0001 | 0.0637 | 0.877 | 0.664 | 1.157 | 1.158 | 1.051 | 1.275 |
| 27 | 34306 | 104 | 34249 | 102 | 602101 | 754 | 579572 | 646 | 0.3694 | <.0001 | 0.5092 | 1.018 | 0.774 | 1.338 | 1.124 | 1.011 | 1.248 |
| 28 | 34261 | 113 | 34212 | 100 | 601916 | 645 | 579420 | 540 | 0.0806 | <.0001 | 0.9027 | 1.129 | 0.862 | 1.478 | 1.150 | 1.026 | 1.289 |
| 29 | 34208 | 111 | 34132 | 92 | 601201 | 522 | 578728 | 452 | 0.0601 | <.0001 | 0.6068 | 1.204 | 0.913 | 1.588 | 1.112 | 0.980 | 1.261 |
| 30 | 34098 | 115 | 33988 | 96 | 600220 | 532 | 577860 | 459 | 0.0595 | <.0001 | 0.6555 | 1.194 | 0.911 | 1.567 | 1.116 | 0.985 | 1.264 |
| 31 | 33969 | 101 | 33821 | 85 | 599184 | 497 | 576937 | 405 | 0.0384 | <.0001 | 0.9930 | 1.183 | 0.886 | 1.580 | 1.182 | 1.036 | 1.347 |
| 32 | 33798 | 100 | 33573 | 80 | 598090 | 513 | 575880 | 422 | 0.0226 | <.0001 | 0.7188 | 1.242 | 0.925 | 1.667 | 1.170 | 1.029 | 1.331 |
| 33 | 33580 | 82 | 33249 | 74 | 596854 | 483 | 574773 | 371 | 0.0675 | <.0001 | 0.4474 | 1.098 | 0.802 | 1.504 | 1.254 | 1.095 | 1.435 |
| 34 | 33282 | 87 | 32844 | 76 | 595523 | 523 | 573548 | 435 | 0.1138 | <.0001 | 0.8863 | 1.130 | 0.830 | 1.538 | 1.158 | 1.020 | 1.315 |
| 35 | 32915 | 85 | 32381 | 74 | 593918 | 539 | 572083 | 455 | 0.1379 | <.0001 | 0.9557 | 1.130 | 0.827 | 1.544 | 1.141 | 1.007 | 1.293 |
| 36 | 32427 | 82 | 31822 | 72 | 591915 | 628 | 570133 | 551 | 0.2341 | <.0001 | 0.9170 | 1.118 | 0.814 | 1.534 | 1.098 | 0.979 | 1.231 |
| 37 | 31816 | 60 | 31120 | 59 | 589115 | 672 | 567607 | 599 | 0.7057 | <.0001 | 0.6650 | 0.995 | 0.694 | 1.425 | 1.081 | 0.968 | 1.207 |
| 38 | 30998 | 45 | 30209 | 48 | 584792 | 748 | 563429 | 664 | 0.9670 | 0.0469 | 0.4206 | 0.913 | 0.608 | 1.372 | 1.085 | 0.978 | 1.205 |
| 39 | 29841 | 32 | 28963 | 35 | 576747 | 661 | 555733 | 588 | 0.8748 | 0.7915 | 0.4273 | 0.887 | 0.549 | 1.434 | 1.083 | 0.969 | 1.211 |
| 40 | 28090 | 24 | 27246 | 23 | 562197 | 510 | 542172 | 470 | 0.8475 | 0.7723 | 0.9111 | 1.012 | 0.571 | 1.794 | 1.046 | 0.923 | 1.186 |
| 41 | 25396 | 12 | 24476 | 11 | 535712 | 287 | 517717 | 268 | 0.8430 | 0.5311 | 0.9705 | 1.051 | 0.464 | 2.383 | 1.035 | 0.876 | 1.222 |
| 42 | 21126 | 8 | 20368 | 4 | 483629 | 140 | 470262 | 144 | 0.3358 | 0.7784 | 0.2532 | 1.928 | 0.581 | 6.404 | 0.945 | 0.749 | 1.193 |
