# Supplementary material for: Sex differences in growth and mortality in pregnancy-associated hypertension
Source: PLoS One. 2024 Jan 11;19(1):e0296853. doi: 10.1371/journal.pone.0296853 (PMC10783718; doi:10.1371/journal.pone.0296853)
Supplement: S2 Fig — Effects of sex and survival on birthweight in White pregnancies (left) and Black pregnancies (right). (DOCX) [file pone.0296853.s007.docx]

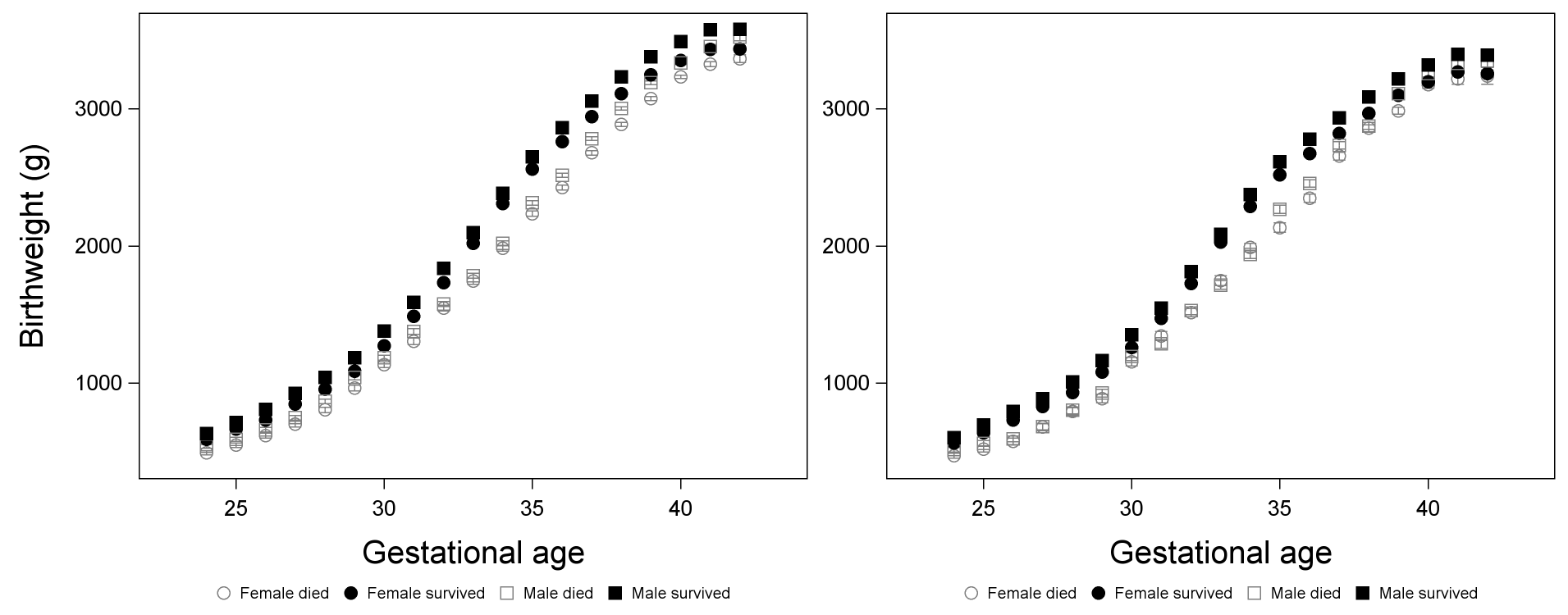


S2 Figure. Effects of sex and survival on birthweight in White pregnancies (left) and Black pregnancies (right). Values are least squares means ± standard errors from a model including gestational age as a categorical variable, sex, survival, group, tobacco use (yes or no), year and all possible pairwise and three-way interactions between gestational age, sex and survival.
