# Supplementary material for: Sex differences in growth and mortality in pregnancy-associated hypertension
Source: PLoS One. 2024 Jan 11;19(1):e0296853. doi: 10.1371/journal.pone.0296853 (PMC10783718; doi:10.1371/journal.pone.0296853)
Supplement: Attachment — Submitted filename: Response to Reviewers.docx [file pone.0296853.s008.docx]

Dear Dr. Daud,

Thank you very much for your response to our manuscript, and to the reviewers for their constructive comments. Below, we have outlined our responses to each of the comments. For clarity, we have included the reviewers’ comments in italics. Line numbers refer to the version of the manuscript with “track changes”.

Sincerely,

Julian

*3. Have the authors made all data underlying the findings in their manuscript fully available?*

*Reviewer #1: No*

*Reviewer #2: No*

The data used in our study is publicly available in a public repository:

<https://www.cdc.gov/nchs/data_access/vitalstatsonline.htm>

We have added a link to the citation in the reference list.

*Reviewer #1: Thank you for inviting to review this manuscript for PLOS One.*

*The topic of the research is of epidemiological and physiology interest only, with no implications for the clinical practice irrespective of the study findings themselves.*

We agree, but respectfully note that implications for clinical practice is not a criterion for publication in PLoS ONE, and that this is unrelated to the soundness of our conclusions.

*Furthermore, there are major weaknesses as below:*

*- The definition of hypertension is too vaque and not consistent with current definitions.*

We have added the definition of pregnancy-associated hypertension provided in the user guides to the data (lines 124-128). Note that the definition used (an increase in blood pressure of at least 30 mm Hg systolic or 15 mm Hg diastolic on two measurements taken 6 hours apart after the 20th week of gestation) is quite similar to the current definition (a blood pressure (BP) of ≥140/90 mmHg on two occasions (at least 4 hours apart) after 20 weeks' gestation in a previously normotensive woman; <https://bestpractice.bmj.com/topics/en-us/663>). We note that it is common for retrospective studies such as ours to use definitions that were in place at the time of data collection, and not necessarily at the time of publication. For example, the following recent study, published in the American Journal of Obstetrics and Gynecology (Impact factor 10.7) examined births between 1989 and 2021, and used ICD codes to identify cases of preeclampsia, and so cases would have been defined at the time of data collection.

<https://www.ajog.org/action/showPdf?pii=S0002-9378%2823%2900382-4>

[*https://doi.org/10.1016/j.ajog.2023.06.010*](https://doi.org/10.1016/j.ajog.2023.06.010)

*- another major limitation to the study findings is represented by tha acknowledged role of maternal haemodynamics in the patophysiology of hypertensive disorders of the pregnancy and on fetal growth. I cannot see one line on the role of maternal haemodynamics and hypertension in pregnancy despite the extensive research so far conducted. I understand this is not of interest for the Authors, but the paper needs such information to be added. Fetal gender is, of course, independent from maternal hemodynamics.*

We added an acknowledgement of the potential role of maternal haemodynamics to the manuscript (line 343-348). However, we do not understand how this represents a limitation to our findings.

*- I cannot see anywhere the number of the overall included cases and the numbers involved in sub-analysis. They all should be added to the text.*

We have added a flow chart showing inclusions/ exclusions and overall sample sizes (Figure 1 in the revision).

*Additional comments*

*- Line 324: the concept of early- and late-preeclampsia is outdated*

We have changed these terms to “preterm” and “term”, to be consistent with the 2020 systematic review that we cite in this section. However, we note that the concept of classifying preeclampsia by timing of onset is not outdated. A 2022 paper in Human Reproduction Update (highest impact factor among Obstetrics and Gynecology journals; https://doi.org/10.1093/humupd/dmac027) uses these terms, as does a 2022 paper in American Journal of Obstetrics and Gynecology (second highest impact factor among Obstetrics and Gynecology journals; <https://www.ajog.org/article/S0002-9378(20)31283-7/fulltext>). We have retained the terms “early-onset” and “late-onset” in a different section (line 292) to be consistent with the source that we are citing.

*- Line 325: according to the latest definition of preeclampsia proposed by the Intenational Society for the Study of Hypertension in Pregnancy (ISSHP) preeclampsia is no longer distinguished into mild, moderate and severe.*

We do not use categories of severity in any of our analyses. The reviewer refers to a statement in our Limitations section, where we acknowledge that “we could not determine the severity of preeclampsia”. While there are not clear categories of severity, some cases of preeclampsia are more severe than others. The ISSHP’s position is that the term ‘mild’ should not be used in clinical practice to avoid creating a false sense of security among clinicians and patients:

“Although ISSHP has formerly published a statement documenting ‘severe pre-eclampsia’, we agree with the position of ACOG and others that pre-eclampsia may become a major threat to mother and baby at any stage and classification into ‘mild’ or ‘severe’ disease can be erroneous or misleading to less experienced clinicians. ACOG has eliminated the diagnosis of ‘severe pre-eclampsia’ and instead discusses ‘Pre-eclampsia with or without severe features’, a sensible clinical approach.”

<https://www.sciencedirect.com/science/article/pii/S2210778918301260?via%3Dihub#b0260>

As the Reviewer has noted, our work is not directed primarily at clinicians, and so there seems to be little danger of misleading this group with this statement.

*Reviewer #2: Sex differences in growth and mortality in pregnancy-associated hypertension.*

*This manuscript puts together interesting empirical tests of the hypothesis that male fetuses prioritize growth in the presence of adversity in contrast to female fetuses who reduce growth. The authors compare male and female mortality and birthweight of fetuses in pregnancies with and without preeclampsia. The authors use public-use Birth Cohort Linked Birth – Infant Death Data Files from the National Center for Health Statistics of the Centers for Disease Control and Prevention from the years 1989-1991, and 1995-2003. While their way of thinking about this hypothesis is interesting, it is difficult to evaluate this manuscript because key pieces of information are not included and the population selection criteria may introduce bias.*

*The authors apply a large number of exclusion criteria for the definition of their population. Most are reasonable.They focus on White and non-Hispanic Black nulliparous women (reasonable) with a singleton pregnancy and valid sex. They also remove states with high numbers of missing data or small samples.*

We thank the Reviewer for recognizing the novelty of our work, and have added the information requested (as described below).

*However, they also set an age restriction which may not make sense since the outcome is associated with age - more justification is needed for this criterion.*

We have added justification for the age restriction (lines 116-118):

“We placed restrictions on maternal age to avoid confounding effects of increased complications at young and advanced ages, e.g., increased rates of stillbirth, fetal growth restriction, small-for-gestational age, preterm birth at advanced maternal age”

Including advanced maternal age pregnancies would have potentially changed associations between low birthweight, preterm birth and pregnancy associated hypertension or stillbirth, not because of fetal growth strategies (the focus of our study), but because of shared association with advanced maternal age. Arguably, fetal growth strategies might change in advanced maternal age, but we did not have any *a priori* predictions regarding such changes, in contrast to differences between the sexes. Smaller sample sizes for advanced maternal age pregnancies would have had reduced statistical power, which could have complicated the interpretation of results (e.g., if an effect was significant in pregnancies 18-34, but not in advanced maternal age).

Limiting our analysis to ages 18-34 strengthens the soundness of our conclusions for pregnancies in this age range. In the Limitations section, we acknowledge that these conclusions may not generalize to other groups (line 334).

*Furthermore, they exclude observations with implausible birthweights for gestational age, defined as those below the 0.5th percentile or above the 99.5th percentile by gestational age and sex. However, as their interest is in cases with potentially very low birthweight, including stillbirths with IUGR, more information on these exclusions are needed.*

In the absence of errors in the dataset, this approach would only exclude 1% of births, and so this would have little effect on the results. Because this exclusion is based on gestational age and sex, it will not lead to bias in our analyses (i.e., PAH pregnancies or early deaths of one sex are not more likely to be excluded than those of the other sex). However, we have provided additional information in a new flow chart which addresses this issue (Figure 1, described in further detail below).

*There was no descriptive table of the sample and I could not find the sample sizes for any of the analyses, so it was not clear how these restrictions affected the sample. In addition to this table, some analysis on the sex and preeclampsia of excluded fetuses is needed. The absence of these tables also prevented the evaluation of whether the data were plausible and how close they are to known incidence of the outcomes being evaluated.*

We have added a flow chart (Figure 1) which clearly shows the inclusions/ exclusions and the sample sizes for the analyses. In particular, we have included the number of cases excluded because of implausible birthweights. Overall, 3.8% of White pregnancies and 5.5% of Black pregnancies were excluded on the basis of birthweight, whereas 1% would be expected given that the top and bottom 0.5% were excluded. This suggests that this step excluded some errors, but the proportions of exclusions were very similar for males and females, indicating that this would not create bias in our analyses. The proportions of pregnancies excluded on the basis of birthweight were greater for PAH (pregnancy associated hypertension) than controls, suggesting that some very severe cases of growth restriction in PAH may have been excluded. However, these represent a small proportion of PAH cases (the difference between controls and PAH is < 4%) and so we believe that it is better to err on the side of caution, i.e., risk excluding some severe cases rather than including erroneous data. Moreover, in both males and females, the proportion of births excluded because birthweight was too high was similar to the proportion excluded because it was too low, i.e., not all of the exclusions involve small babies. This suggests that many of the errors, including some of the implausibly low birthweights, are due to errors in the estimation of gestational age.

*Other selection decisions may also induce bias. The first analysis starts by looking at differences in birthweight, but only among births surviving to 29 days. This would exclude severe cases of preeclampsia and deaths related to IUGR. If there is differential mortality by sex (which is one of the hypotheses), this could affect this analysis.*

The exclusion of fetal deaths from our test of Prediction 1 would exclude the most severe cases of PAH and/ or IUGR, which might be more prevalent in one sex. However, this possibility is addressed in the tests of Predictions 2 and 3, and so this “bias” is addressed. However, the approach used for Prediction 1 does not introduce bias, but rather addresses a specific prediction (ie, “Among surviving fetuses, differences in birthweight between PAH and controls will be greater among females than among males”). We have described our rationale for this approach in the manuscript (i.e., “to exclude infants whose weight may have been influenced by a major congenital abnormality”, line 147). Because major abnormalities unrelated to PAH might also differ in prevalence between the sexes, we believe this exclusion is justified despite the exclusion of severe cases of PAH.

*In general, in the absence of descriptive tables, it was difficult to follow the analyses. The graphs were very difficult to read and interpret because they present a lot of information with overlapping confidence intervals and large scales. The multivariable models were not possible to interpret without baseline information.*

We believe that the Reviewer is referring primarily to what was Figure 4 in the original submission (now Figure 5 in the revision), which does have large, overlapping confidence intervals. We have therefore added supplementary tables which presents the numbers underlying this figure, as well as the results of statistical tests (S3 Table and S4 Table). We include them as supplementary tables as they are somewhat redundant with the figure.

Figures 2 and 3 present differences in birthweight (between PAH pregnancies and controls or between infants that died and those that survived, respectively) against gestational age for males and females separately, and we cannot think of how to present this more clearly; many numbers in a table would certainly be more difficult to follow. Figure 4 presents mortality rate against gestational age for four groups, and again we cannot think of how to present this more clearly. Figures 6 and 7 address sex differences in the odds of birth, and so do not address our predictions, but provide some context that many readers will seek.

*I would suggest that the authors provide full descriptive tables of their exposure and outcomes and consider showing some of the data now in graphs as figures.*

We have added a flow chart (Figure 1), as described above. We believe that the Reviewer meant “showing some of the data now in graphs as tables”, and so have done this for Figure 5, as described above.

*One issue to consider is that preterm births for preeclampsia and IUGR mainly occur if the fetus is detected as having a problem and is an indicated birth. This information is important for understanding these relationships because most fetal biometric charts used to interpret ultrasound measures are not sex-specific. Therefore, more female fetuses may be detected and also have resulting clinically decided preterm births. This information (prelabor cesarean) should be part of the information provided in descriptive tables.*

We agree that some of the preterm births will have been indicated because of preeclampsia and/ or IUGR. However, this does not affect the interpretation of our results. Whether a birth was indicated or spontaneous would not affect birthweight at a given gestational age (the focus of predictions 1 and 2), although fetal size (and hence birthweight) might influence whether birth was induced, which we believe is what the Reviewer is suggesting. The latter effect would be unlikely to influence the former, but also would be unlikely to be influenced by fetal sex. In the case of preeclampsia, indicated delivery would likely be based on maternal factors (e.g., blood pressure) or Doppler measures rather than fetal size. Similarly, in IUGR, the decision to deliver is likely influenced by Doppler velocimetry and/or fetal growth trajectory, and not simply on a static measure of fetal size, e.g., see

https://pubmed.ncbi.nlm.nih.gov/21962625/

https://pubmed.ncbi.nlm.nih.gov/14770388/

https://pubmed.ncbi.nlm.nih.gov/12380678/

We have deliberately referred to older papers (2002-2011), as these will more closely reflect the thinking at the time of births in the study. In summary, the possibility that females might be more likely to be delivered because they are normally smaller would be unlikely to have a large influence on analyses.

Indicated birth may have improved survival (tested in prediction 3), but as described above, it seems unlikely that females would benefit from this disproportionately. Prediction 3 is that "The difference in mortality between the sexes will be higher among pregnancies affected by PAH" and so, in order to influence our results, females would not only need to benefit disproportionately from indicated birth; the disproportionate benefit would have to be greater in one group (PAH or controls) than the other.

The dataset does indicate mode of delivery, but not whether labour occurred prior to a Cesarean. At any rate, even if we could report rates of prelabour Cesareans, a difference between the sexes could indicate a difference in thresholds for delivery between the sexes (i.e., what we think the Reviewer is suggesting), or a real sex difference in the rate of complications necessitating an emergency Cesarean.
