# Supplementary material for: Sex differences in growth and mortality in pregnancy-associated hypertension
Source: PLoS One. 2024 Jan 11;19(1):e0296853. doi: 10.1371/journal.pone.0296853 (PMC10783718; doi:10.1371/journal.pone.0296853)
Supplement: Attachment — Submitted filename: Response to reviewers.docx [file pone.0296853.s009.docx]

Dear Dr. Daud,

Thank you very much for your response to our manuscript, and to the reviewers for their constructive comments. Below, we have outlined our responses to each of the comments. For clarity, we have included the reviewers’ comments in italics. Line numbers refer to the version of the manuscript with “track changes”.

Sincerely,

Julian

Reviewers' comments:

*2. Is the manuscript technically sound, and do the data support the conclusions?*

*Reviewer #2: Partly*

*Reviewer #3: No*

We believe that Reviewer 2’s concern relates to the exclusions. We have clarified this issue and provided more detail, but it remains unclear to us how this issue might undermine our conclusions.

We believe that Reviewer 3’s concern regarding our support for the conclusions is due to a confusing statement in the abstract that has been clarified.

*3. Has the statistical analysis been performed appropriately and rigorously?*

*Reviewer #2: Yes*

*Reviewer #3: No*

We believe that Reviewer 3’s assessment is due to a few instances where p-values between 0.05 and 0.1 were referred to as marginally non-significant. We have removed such terminology. None of our conclusions relied on these results. Instead, in our previous manuscript, we had attempted to err on the side of caution by acknowledging that some of these “marginally non-significant” might be real patterns. Our conclusions are actually strengthened by considering them to be not significant.

*5. Is the manuscript presented in an intelligible fashion and written in standard English?*

*Reviewer #2: Yes*

*Reviewer #3: No*

Beyond the confusing statement in the abstract, there are no reviewer comments related to the quality of the writing.

*Reviewer #2: Thank you to the authors for their attention to the comments in the first round of reviews.*

*I remain concerned about the exclusions, which may impact on the results since they may exclude many growth restricted fetuses (which is a focus of the paper). The authors remove births with BW<.05 percentile. However, they use birthweight charts which are well known to be biased for preterm births (the charts are constructed from births, many of whom are growth restricted).*

We believe that when Reviewer #2 refers to birthweight charts as “biased”, they mean that the charts are not representative of fetuses *in utero*; for preterm gestational ages, the charts are constructed from those who are born preterm, and thus there is an overrepresentation of growth restricted fetuses, compared to fetuses who continued to grow *in utero*. In this sense, this bias will reduce the 0.5^th^ percentile, such that we will include smaller fetuses than we would if we used “correct” percentiles for all fetuses (born and not born at a given gestational age). This reduces the concern that this exclusion is excluding growth restricted fetuses with truly low birthweights.

Importantly, the birthweight percentiles that we used (from ref 39) were calculated using the same database, albeit from a different year (2017 vs 1989-2003 in our study), i.e., to the extent that the birthweight percentiles are “biased”, it is expected that the percentiles are biased in the same way and to the same extent as our birthweight data. Crucially, this “bias” would affect both males and females, and it is unclear how it could compromise our results.

We also note that, in the absence of errors in the dataset, this approach would only exclude 1% of births, and so this would have little effect on the results. Inevitably in datasets of this size, there are some errors, and we need an objective approach to remove implausible values. An alternative would be to use growth curves of estimated fetal weight based on ultrasound measurements (e.g., DOI:10.1371/journal.pmed.1002220), but these would suffer other limitations (e.g., constructed using a different population, which might have different birthweights; errors in the estimation of weight from ultrasound measurements; assumption that same formula can be used to calculate estimated fetal weight from ultrasound measurements in males and females, etc.).

*This will be true in particular for births with fetal growth restriction. The authors should compare their exclusions, not just on the full population, but on their outcomes - the numbers excluded by gestational age for live and stillbirths by sex and by group. It is possible that there are many more exclusions in these high risk groups. The authors note that they have an equivalent proportion of exclusions at higher birthweights, but GA errors are much more likely to be in the direction of term babies being improperly coded as preterm (i.e. 38 as 28, given so many more 38 weekers, for instance), so having more high birthweights would be expected.*

We have added supplementary tables including the numbers and percentages of observations excluded based on birthweight, separately by gestational age, sex, group, and whether the exclusion was because birthweight was too low or too high. At the bottom of the tables, we have also summarized exclusions by WHO gestational age category (Term: 37 weeks or greater; Moderately preterm: 32-36 weeks; Very preterm: 28-31 weeks; Extremely preterm: less than 28 weeks).

Importantly, exclusion rates are not consistently different between males and females, and so are unlikely to create biases in our comparisons or compromise our conclusions, which focus on differences between males and females. We do not compare stillbirths and livebirths, or compare PAH and controls; we compare whether the differences between these groups differs between the sexes. Thus, even if there is a potential bias in the comparison of PAH and controls (e.g., because the number of accurate observations that are excluded for PAH is higher than that for controls), this bias will be present for both males and females, and so will not create or obscure an interaction between sex and group. The interactions between sex and group (or lack thereof) form the basis of our conclusions.

Fetal deaths show higher proportions of pregnancies that are excluded because birthweight is too low. However, the exclusion of these pregnancies is not necessarily erroneous or undesirable. In some cases, the fetus will have died well before birthweight was recorded, such that the gestational age, and potentially the birthweight, may not reflect the age and weight at death. Exclusion of such pregnancies would therefore be desirable and not an error. The absolute numbers of excluded fetal deaths is small.

From our original submission, we have been very transparent about our quality control procedures. There are some implausible values in the dataset (e.g., birthweights below 100 g at 24 weeks or later), and we need an objective procedure to exclude these. We believe that our approach, while imperfect, is the best solution and does not compromise the soundness of the work, as explained above.

On a more minor note, during these revisions, we noticed an omission in Fig. 1, which is that the numbers of excluded observations include not only those excluded because birthweights were below the 0.5^th^ percentile or above the 99.5^th^ percentile, but also those excluded because birthweight was missing. This omission has been corrected in Fig. 1. The supplementary table includes only those excluded because birthweights were below the 0.5th percentile or above the 99.5th percentile, thus reducing redundancy with the figure.

*It would also be nice to have an introductory table with overall descriptive data for the two groups and sex, the distribution by GA, the covariables and mortality.*

We have added a table with summary data. We have presented the number of pregnancies by gestational age according to WHO categories.

*I am not sure why the authors use a sliding scale for their fetus at risk calculations - this makes it difficult to compare the results with other samples since this is not the usual practice.*

We used a sliding scale because in some groups, the sample sizes become very small in a given gestational week. Consequently, the results can vary substantially from one week to the next due to sampling error, which makes both the interpretation and visualization more difficult. The sliding scale smooths out such variation. We note that some other studies do not encounter this difficulty because they use a proportional hazards model, which “smooths” out the data but fitting a function across gestational ages. However, as described in our results, we found the proportional hazards assumption to be violated, and so instead performed logistic regression at different time points. K.S. Joseph, who has authored some fetus-at-risk studies suggested that our approach was reasonable (personal communication).

If readers wish to compare our results with those of previous studies, the numbers by gestational week are provided in S3 Table and S4 Table.

*Also, do the adjustments change the OR or differences in BW? It is not possible to know this from the data presented.*

It is not clear which adjustments the Reviewer is referring to. Apart from the terms that are the focus of the study (sex, group, and survival) and gestational age, the only covariates in the analyses of birthweight are tobacco use and year. Given the significance of these covariates, the value of reporting unadjusted birthweight is not clear.

Odds ratios for the analyses of death and birth include only effects of sex, group and the interaction between sex and group (as described in manuscript). Thus, the OR are not adjusted for anything other than the effects we are testing. It is not clear what unadjusted OR would be relevant.

*Finally, if the authors are worried about congenital anomalies and birthweight, wouldn't it make sense to remove those with congenital anomaly codes only? Also, this problem would also be present for the mortality analysis, right?*

We are not worried about specifically about congenital anomalies; we have reworded and removed mention of congenital anomaly to avoid confusion. Neonatal deaths are defined as deaths within the first 28 days of life, and drop off sharply within this time period (e.g., <https://www.ncbi.nlm.nih.gov/pmc/articles/PMC9794155/>), i.e., if the infant is going to die, it is highly likely to die within this period. This is therefore an objective and reasonable cut-off to use for our analyses of “surviving fetuses”.

*If the authors want to do their analysis on survivors after 28 days, they might consider presenting the mortality analyses first since this respects the time sequence of events and may be less confusing.*

We cannot see a potential source of confusion in the order of these sections. Note that our three predictions are:

Prediction 1: Among surviving fetuses, differences in birthweight between PAH and controls will be greater among females than among males

Prediction 2: Among all fetuses, differences in birthweight between fetuses that survive and those that die prenatally or shortly after birth will be greater among females than among males

Prediction 3: The difference in mortality between the sexes will be higher among pregnancies affected by PAH

We do see the Reviewer’s argument for switching Predictions 1 and 2, i.e., comparing those who survive with those who do not (Prediction 2) and then examining those who survive (Prediction 1). However, the order would then be an analysis of mortality (Prediction 2), an analysis of survivors (Prediction 1), and then another analysis of mortality (Prediction 3), which could also be considered problematic.

These are three separate predictions and there is not a logical sequential order to them, i.e., one does not logically follow another.

*Reviewer #3:*

*1) Method*

*Line 124 : Have the authors considered gestational diabetes as a confounding factor? GDM can affect the fetal weight*

The data available does not distinguish between gestational diabetes and preexisting diabetes, and does not provide any information regarding how diabetes was managed (which would be critical in consideration of its effects on birthweight). While GDM would certainly be expected to affect birthweights, it is not clear how it might be a confounding effect in our study. There is some evidence that pregnancies with a male fetus are at greater risk of GDM, which might contribute to differences between males and females, but it is not clear how it would confound analyses of differences between PAH and non-PAH pregnancies, or between surviving and non-surviving fetuses.

*2) Result*

*Line 229 : P=0.08 is non-signifcant, not marginally non-significant*

*The sentence is confusing 'marginally non-significant or significant'.. especially due to the choice of word*

*'or'. Please rephrase this sentence.*

We have removed mention of the marginally non-significant result, which clarifies the sentence.

*Line 232 : Stating the P value as P<0.1 is inadequate and cannot be regarded as significant. Please state the actual P value, as the value from 0.05 - 0.1 is huge and this value determines the significance of the*

*finding.*

We have removed mention of the marginally non-significant result.

*3) Discussion*

*Line 289 - 290 : 'A previous study....' This statement agrees with the study's result. But the study's result was contradictory to the hypothesis. Please discuss the reasons why the finding of the study was*

*inconsistent with the hypothesis.*

We have added a potential explanation of our finding: “While not consistent with our prediction, this result might indicate that pregnancies with male fetuses are associated with more severe forms of PAH/ preeclampsia, although we did not have information regarding the severity of the condition.”

*Line 301 : 'However we found the opposite.' The author did not discuss the possible reasons as to why the restrictions in the new cohort vs. non-restrictions in the previous cohort influenced the result.*

We have added the following potential explanation: “The additional restrictions in the present study may have reduced confounding effects (e.g., differences in the prevalence of complications) and provided more accurate patterns. Alternatively, births in the previous cohort [36] occurred from 1959 to 1966 whereas those in the present study occurred from 1989 to 2003, and so changes in clinical management may have led to real changes in patterns of birthweight and survival.”

*Line 312 - 314 : Please discuss the possible reasons why higher deaths in control vs PAH pregnancies in early gestation.. this is in order to address possible confounding factors that had caused this result*

To clarify, PAH pregnancies had higher mortality than control pregnancies throughout gestation until around term (Fig. 3). The actual difference is “Early in gestation, males had a higher risk of death in control pregnancies than females, but a lower risk in PAH pregnancies, inconsistent with our prediction.” We have added the following potential explanation: “This reversal of sex differences in PAH pregnancies may indicate that females are exposed to more severe PAH at very early gestational ages (weeks 24-26).”

*Line 316 - 318: This statement implies that sex does not influence mortality in PAH pregnancies. But the conclusion in the Abstract (line 37 - 39) is contradictory to this statement (further details below).*

We apologise for the confusion due to our wording in the abstract. Our intended meaning was that few of the differences were consistent with the hypothesis, i.e., there was little support for the hypothesis. We have reworded to clarify, which removes the perceived contradiction.

*4) Conclusion*

*Line 360 - 368 : These statements clearly stated that the results did not support the growth theory hypothesis. But the Conclusion in the Abstract stated differently and is misleading to the whole content of the study.*

As described above, the wording of the abstract has been clarified, and so the results, discussion, conclusion and abstract are all consistent.
